# Clinical characteristics of patients with early-onset diabetes mellitus: a single-center retrospective study

**DOI:** 10.1186/s12902-023-01468-2

**Published:** 2023-10-10

**Authors:** Wenjing Dong, Saichun Zhang, Shiju Yan, Zhizhuang Zhao, Zengqiang Zhang, Weijun Gu

**Affiliations:** 1 Chinese PLA Medical College, Beijing, 100039 China; 2 Department of Endocrinology, The first medical center of Chinese PLA General Hospital, Beijing, 100853 China; 3 Department of Gerontology, Hainan Hospital of Chinese PLA General Hospital, Sanya, 572013 Hainan China; 4 Department of Orthopedics, Hainan Hospital of Chinese PLA General Hospital, Sanya, 572013 Hainan China

**Keywords:** Early-onset diabetes mellitus, Fulminant type-1 diabetes mellitus, Latent autoimmune diabetes in adults, Risk factors, Microangiopathy, Diabetic ketoacidosis

## Abstract

**Background:**

The prevalence of diabetes mellitus (DM) is dramatically increasing around the world, and patients are getting younger with changes in living standards and lifestyle. This study summarized and analyzed the clinical characteristics of different types of newly diagnosed diabetes mellitus patients with an onset age between 18 and 40 years to provide clinical evidence for the early diagnosis and treatment of diabetes, reduce short-term and long-term complications and offer scientific and personalized management strategies.

**Methods:**

A total of 655 patients newly diagnosed with early-onset diabetes mellitus in the Department of Endocrinology, the First Medical Center of PLA General Hospital from January 2012 to December 2022 were retrospectively enrolled in this study, with an onset age of 18–40 years. Their clinical data were collected and investigated. All patients were divided into two groups according to whether they presented with diabetic microangiopathy. Similarly, patients with early-onset type-2 diabetes were grouped in accordance with whether they had ketosis at the time of diagnosis. Binary logistic regression analysis was performed to analyze risk factors, and receiver-operating characteristic (ROC) analysis was used to explore the predictive value of significant risk factors.

**Results:**

The findings were as follows: (1) Of 655 enrolled patients, 477 (72.8%) were male and 178 (27.1%) were female, with a mean age of onset of was 29.73 years ± 0.24 SD. (2) The prevalence of early-onset diabetes was gradually increasing. Type-2 diabetes was the most common type of early-onset diabetes (491, 75.0%). The ages of onset of early-onset type-1 diabetes, type-2 diabetes and LADA were mainly 18–24 years, 25–40 years and 33–40 years, respectively. (3) Initial clinical manifestations of early-onset diabetes were classic diabetes symptoms (361, 55.1%), followed by elevated blood glucose detected through medical examination (207, 31.6%). (4) Binary logistic regression analysis suggested that high serum uric acid (UA), a high urinary albumin-to-creatinine ratio (UACR) and diabetic peripheral neuropathy (DPN) were risk factors for microangiopathy in early-onset diabetes patients (P < 0.05). The area under the curve (AUC) on ROC analysis of the combination of UA, UACR and DPN was 0.848, 95% CI was 0.818 ~ 0.875, sensitivity was 73.8% and specificity was 85.9%, which had higher predictive value than those of UA, UACR and DPN separately. (5) Weight loss, high glycosylated hemoglobin (HbA1c) and young onset age were risk factors for ketosis in patients with early-onset type-2 diabetes (P < 0.05).

**Conclusion:**

(1) Men were more likely to have early-onset diabetes than women. (2) Early-onset diabetes patients with high serum uric acid levels, high UACRs and peripheral neuropathy were prone to microangiopathy. Comprehensive evaluation of these risk factors could have higher predictive value in the prediction, diagnosis and treatment of microvascular lesions. (3) Patients with weight loss at onset, high HbA1c and young onset age were more likely to develop ketosis. Attention should be given to the metabolic disorders of these patients.

## Introduction

Diabetes mellitus (DM) is a type of chronic metabolic disease characterized by disorded carbohydrate, fat and protein metabolism and is caused by the interaction of genetic and acquired factors. In recent years, with the improvement of living standards, changes in lifestyle and an increase in the obesity rate, DM is becoming more prevalent, and patients are getting younger worldwide, which has become an important public health issue affecting human health [1, 2]. Some studies [3, 4] have found that the incidence of type-2 diabetes mellitus (T2DM) now exceeds that of type-1 diabetes mellitus (T1DM) among young people in some Asian regions. A large cross-sectional study [5] in China showed that the prevalence of DM is approximately 5.7% in adults aged < 40 years. Several studies [4, 6, 7] reported that patients with early-onset diabetes mellitus have a higher risk of microvascular and macrovascular diseases, heart failure, and shorter life expectancy than those with late-onset diabetes. Unfortunately, patients with early-onset diabetes are usually busy with their career, relatively healthier than elderly individuals and present no obvious symptoms, leading to a low rate of medical treatment, poor medication compliance and gradual aggravation of pancreatic β-cell dysfunction [8]. Meanwhile, due to the early onset age, long durations of hyperglycemia, multiple complications and other factors, serious physical and mental health issues easily occur in patients, casting a heavy economic burden on families and society. Therefore, this study conducted a single-center retrospective analysis of different types of newly diagnosed hospitalized diabetes mellitus patients with an onset age between 18 and 40 years in Beijing and explored their clinical characteristics to provide evidence for the prevention and treatment of EODM, comprehensive management of metabolic complications and improvement of quality of life.

## Patients and methods

### Study subjects

**Fig. 1:**
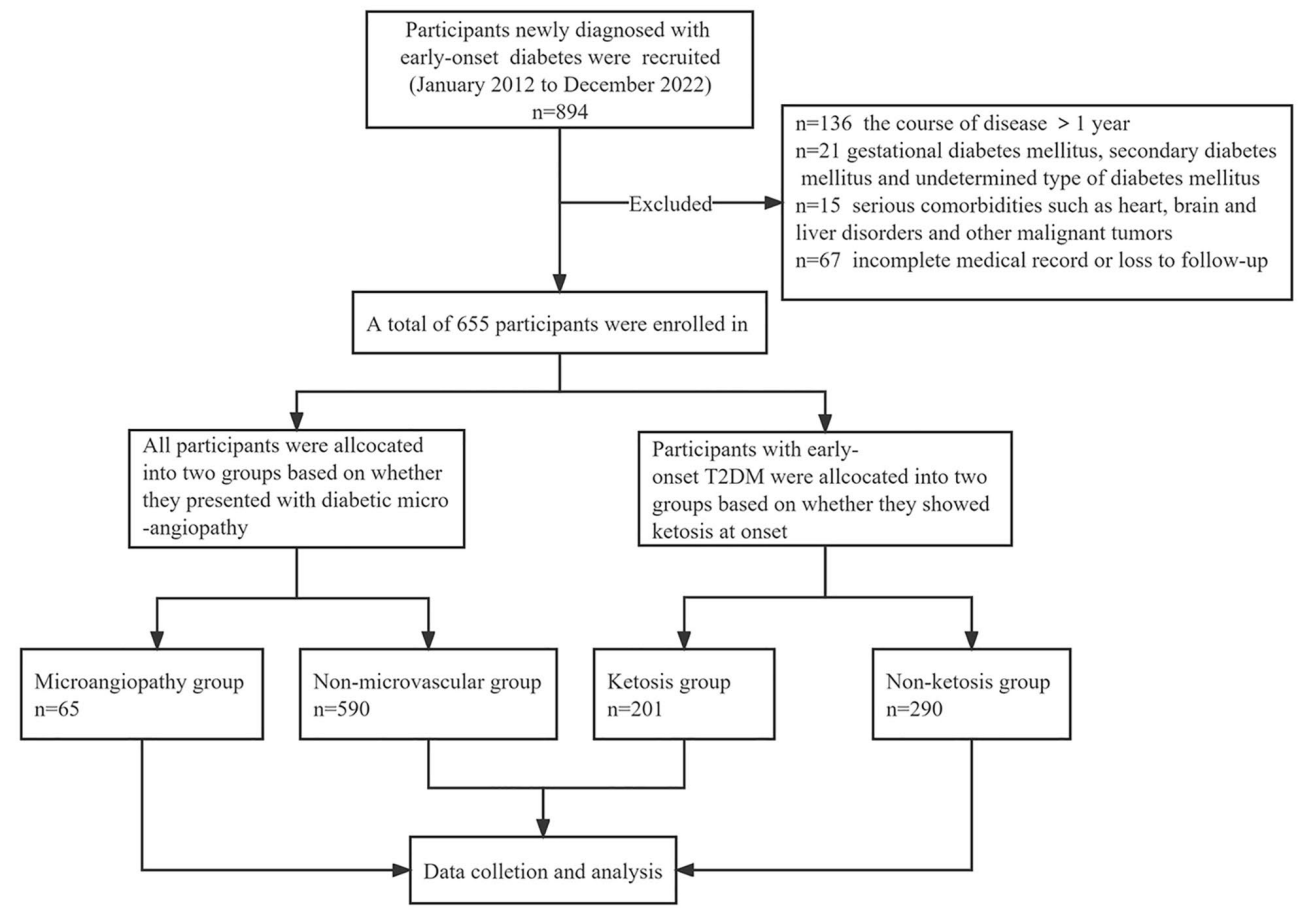
Flow chart of the study design

A total of 894 consecutive patients newly diagnosed with EODM in the Department of Endocrinology, the First Medical Center of PLA General Hospital from January 2012 to December 2022 were involved in this study. Data were manually abstracted by two investigators (DW, YS) from the electronic medical records. This study was approved by the Ethics Committee of Chinese PLA General Hospital. The inclusion criteria were as follows: (1) 1999 World Health Organization (WHO) diagnosis criteria of diabetes [9] and (2) onset age between 18 and 40 years and the course of diabetes less than 1 year. The exclusion criteria were as follows: (1) gestational diabetes mellitus, secondary diabetes mellitus and undetermined type of diabetes mellitus, (2) serious comorbidities such as heart, brain and liver disorders and other malignant tumors, and (3) incomplete medical records or loss to follow-up. Finally, a total of 655 participants were included in our study. See Fig. 1 for details.

### Criteria of diagnosis

1. Latent autoimmune diabetes in adults (LADA) was defined as follows: Consensus on the diagnosis and treatment of LADA recommended by the Chinese Diabetes Association: age of onset ≥ 18 years; positive islet autoantibodies (GADAb, IAA and zinc transporter 8 autoantibody (ZnT8A)), any one or more autoantibodies were positive; and no dependence on insulin therapy for at least half a year after the diagnosis of DM.
2. Keto-prone type-2 diabetes was defined as follows: duration of diabetes ≤ 1 year; and ketosis (blood glucose > 13.9 mmol/L, blood ketone > 0.6 mmol/L or urine ketone body (+) or above) occurred without obvious inducement (infection, surgery, trauma, etc.) [10].
3. Microangiopathy was defined as follows: diabetic nephropathy: urinary albumin excretion ≥ 30 mg/g [11, 12] and diabetic retinopathy: International Clinical Classification of Diabetic Retinopathy (ICDR) [13].
4. Diabetic peripheral neuropathy (DPN) was defined as follows: diagnosed with DM any one or more symptoms and signs of DPN: decreased or absent ankle reflexes, prickling or burning pain predominantly in the feet or legs, and a symmetric decrease in distal sensation and with or without abnormality in nerve conduction. Neuropathy caused by any other diseases was excluded [14].
5. Macrovascular diseases were defined as follows: vascular intimal thickening, atherosclerosis or occlusion confirmed in the carotid artery or lower-extremity artery by ultrasound scan [15].
6. Living habits were defined as follows: according to the WHO definition: smoking more than one cigarette per day for more than 6 months; and alcohol intake > 40 g/d in women and > 80 g/d in men for more than 5 to 10 years.
7. Family history of diabetes mellitus was defined as follows: history of DM in first-degree relatives.
8. Ketosis occurring in the early stage was defined as follows: Blood ketones ≥ 0.6 mmol/L or urine ketones ≥ ++ [10].
9. Nonalcoholic fatty liver disease was defined as follows: diagnosis criteria of nonalcoholic fatty liver disease [16].
10. Hypertension was defined as follows: systolic blood pressure ≥ 140 mmHg and/or diastolic blood pressure ≥ 90 mmHg or hypertension was diagnosed and treated.

### Study parameters

1. General data: Sex, birthplace, age of onset, age of first treatment, time from onset to first hospitalization, initial symptoms, classification of early-onset diabetes, smoking status, marriage status, family history of DM, hypertension, height, weight, body mass index (BMI), weight loss at onset, macrovascular diseases, nonalcoholic fatty liver, microangiopathy, and DPN.
2. Laboratory test results: After fasting for 8–10 h, venous blood samples were collected from all participants in the morning, and biochemical indicators such as fasting blood glucose (FBG), fasting insulin (FIns), 2-h insulin, fasting C-peptide (FCP), 2-h C-peptide, glycosylated hemoglobin (HbA1c), triglyceride (TG), total cholesterol (TC), high-density lipoprotein cholesterol (HDL-C), low-density lipoprotein cholesterol (LDL-C), serum creatinine (SCr), uric acid (UA), alanine aminotransferase (ALT), aspartic transaminase (AST), and gamma-glutamyl transpeptidase (GGT) were measured by an automatic biochemical analyzer in a standard laboratory. The urinary albumin-to-creatinine ratio (UACR) was determined using morning urine.

### Pancreas islet function

This was assessed by the following indicators: with the help of the Homeostasis model assessment 2 (HOMA2) calculator from www.Ocdem.ox.ac.k, islet β-cell function index (HOMA2-%β), insulin resistance index (HOMA2-IR) and insulin sensitivity index (HOMA2-%S) were calculated using FCP for patients treated with insulin and FIns for patients without insulin treatment, and the percentage of C-peptide was used when patients showed FCP < 0.6 ng/mL and FIns < 2.9 mU/L.

### Grouping

1) All newly diagnosed EODM patients were divided into a microangiopathy group and a nonmicroangiopathy group according to whether they presented with diabetic microangiopathy.

2) Patients with early-onset T2DM were divided into a ketosis group and a nonketosis group in accordance with whether they showed ketosis at onset.

### Statistical analysis

Statistical analyses were conducted using SPSS 26.0 (IBM SPSS, USA). Normally distributed data are described as the mean ± SD. Independent samples t test or ANOVA were used to analyze differences between groups. Asymmetrically distributed data are described as the median and interquartile range M (QL, QU), and the Mann-Whitney U test was used to analyze differences between groups. Categorical variables are described as numbers with percentages n (%) and were compared with the χ^2^ test. Binary logistic regression analysis was used for multivariate analysis to determine the risk factors. A receiver-operating characteristic (ROC) curve was subsequently drawn on the basis of predictive factors, and sensitivity and specificity were evaluated using the area under the curve (AUC) value. All analyses used were two-tailed with significance set at p < 0.05.

## Results

### Comparison of general data

**Fig. 2:**
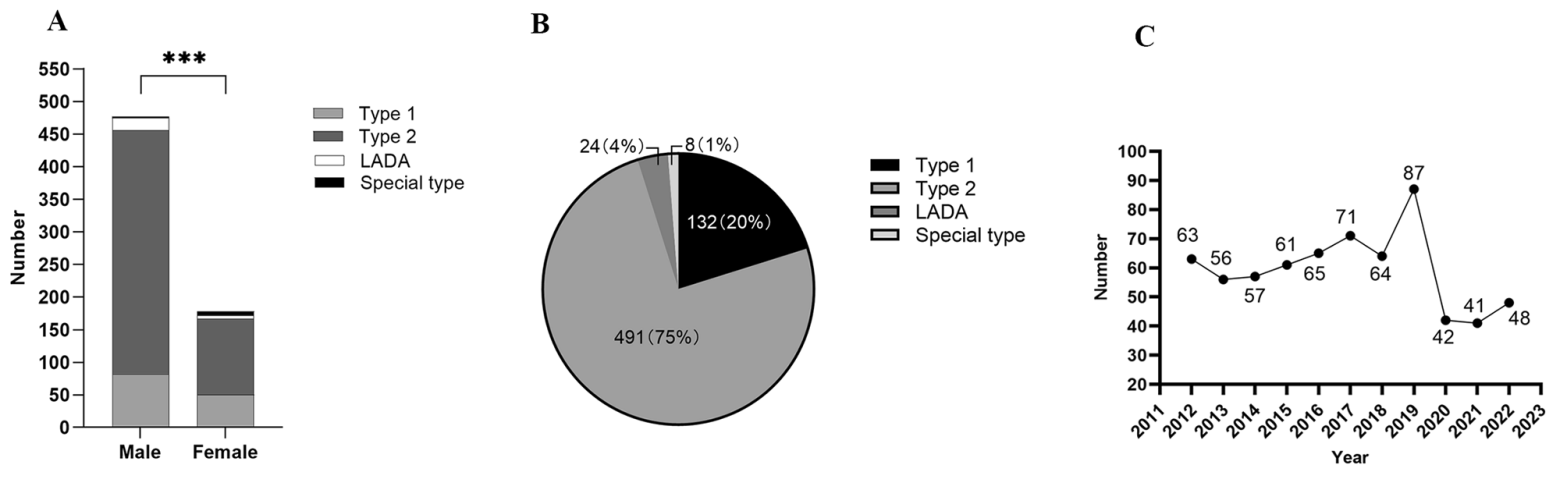
Epidemiological data of EODM patients. (**A**) Males were significantly more likely to have EODM than women. Among male patients, 82 had T1DM, 374 had T2DM, 19 had LADA and 2 had special types. Among female patients, there were 50 patients with T1DM, 117 patients with T2DM, 5 patients with LADA and 6 patients with special types. ***P < 0.001). (**B**) Percentages of different types of EODM in all patients. (**C**) Line chart of the number of patients diagnosed with EODM from 2012 to 2022

**Fig. 3:**
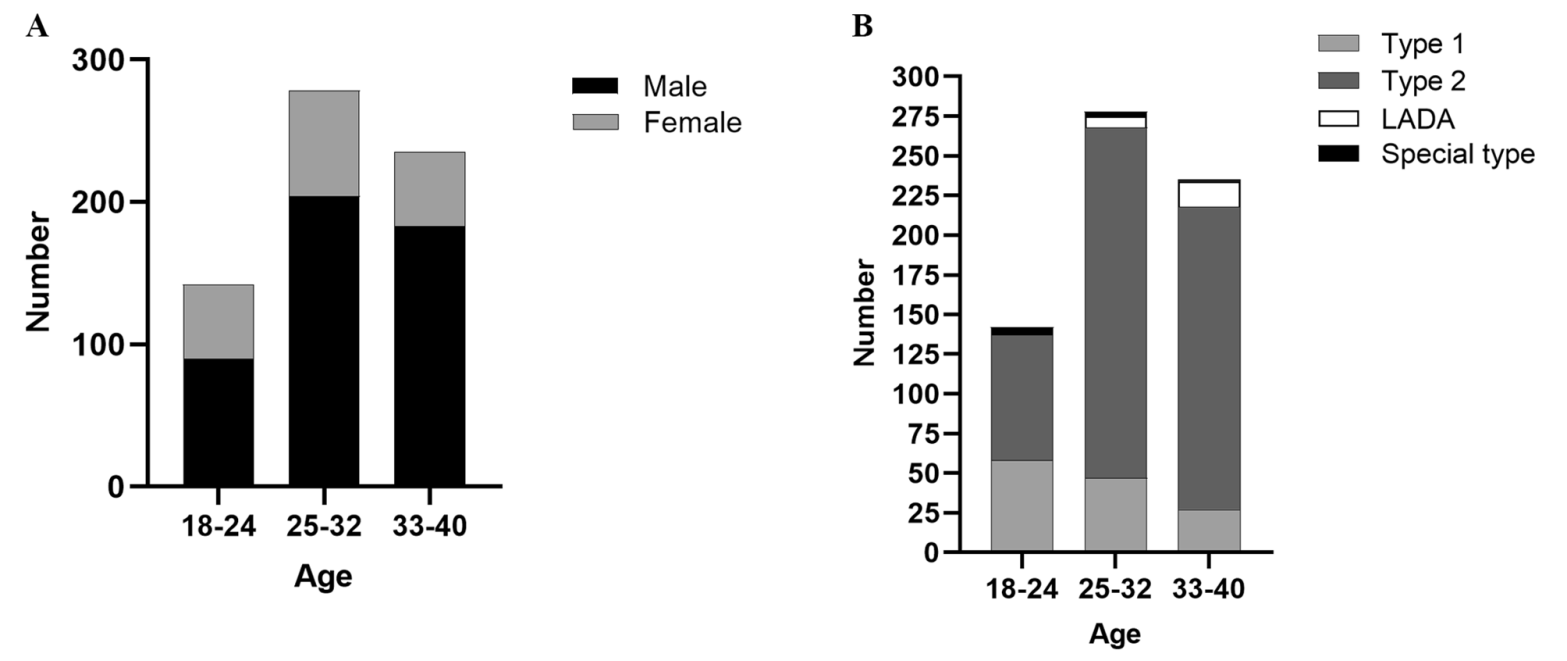
General data of EODM patients (**A**) Percentages of male and female EODM patients in three different age groups. In the 18-24-, 25-32-, 33-40-year-group, there were 90 (63.4%) male EODM patients and 52 (36.6%) female EODM patients, 204 (73.4%) males and 74 (26.6%) females, and 183 (78.5%) males and 52 (21.5%) females, respectively). (**B**) Percentages of different types of EODM in three different age groups. (In the 18-24-year-group, 58 (40.8%) had T1DM, 79 (55.7%) had T2DM, 1 (0.7%) had LADA and 4 (2.8%) had special types. In the 25-32-year-group, 47 (16.9%) had T1DM, 221 (79.5%) had T2DM, 7 (2.5%) had LADA and 3 (1.1%) had special types. In the 33-40-year-group, 27 (11.5%) had T1DM, 191 (81.3%) had T2DM, 16 (6.8%) had LADA and 1 (0.4%) had special types

1. A total of 655 patients were included in this study including 477 (72.8%) males and 178 (27.1%) females. The mean onset age for all patients was 29.73 years ± 0.24 SD (Fig. 2A**)**. Of all patients, there were 132 patients (20.0%) with T1DM, 491 patients (75.0%) with T2DM, 24 patients (4.0%) with LADA and 8 patients (1.0%) with special types of DM, with an average onset age of 26.27 years ± 0.53 SD, 30.55 years ± 0.26 SD, 33.38 years ± 0.91 SD and 25.63 years ± 1.98 SD respectively. Eight special types of DM included 3 cases of mitochondrial diabetes, 2 cases of maturity onset diabetes in young (MODY), 2 cases of steroid-induced diabetes and 1 case of fibrocalcific pancreatic diabetes (Fig. 2B**)**.

2) From 2012 to 2019, the number of patients diagnosed with EODM each year increased and plunged dramatically in 2020 and 2021 (Fig. 2C**)**.

3) In our study, 142 (21.7%) patients, 278 (42.4%) patients and 235 (35.9%) patients were aged 18–24 years, 25–32 years and 33–40 years, respectively. For each group, male and T2DM patients were predominant. The highest proportion of T1DM (58, 40.8%) was observed in the 18-24-years-group, and the 33-40-years-group had the highest proportion of T2DM (191, 81.3%) (Fig. 3**)**.

### Comparison of initial symptoms

Patients with classic diabetes clinical symptoms (polydipsia, polyuria, polyphagia and weight loss) at onset accounted for the largest proportion (361, 55.1%), followed by elevated blood glucose found in medical examination (207, 1.6%), acute ketoacidosis (abdominal pain, fatigue, malignant vomiting, dizziness, fever, etc.) (44, 6.7%), weight loss alone (31, 4.7%) and atypical symptoms (hypoglycemia, blurred vision, fatigue) (12, 1.8%). These 12 atypical symptoms were all found in T2DM (Table 1**)**.

**Table 1:** Onset symptoms in patients with early-onset diabetes

Symptoms of onset	T1DM		T2DM		LADA		Special types	
	Number	N (%)	Number	N (%)	Number	N (%)	Number	N (%)
**Polydipsia, polyuria, polyphagia and weight loss**	97	73.5	243	49.5	17	70.8	4	50
**Emaciation only**	5	3.8	25	5.1	1	4.2	0	0
**Elevated blood glucose**	6	4.5	192	39.1	5	20.8	4	50
**Symptoms of acute ketoacidosis, (abdominalgia, weakness, nausea emesis, dizziness, hypersomnia, obnubilation, fever, etc.)**	24	18.2	19	3.9	1	4.2	0	0
**Other symptoms, (hypoglycemic-like symptom, blurred vision, weak, etc.)**	0	0	12	2.4	0	0	0	0

T1DM: Type-1 diabetes mellitus, T2DM: Type-2 diabetes mellitus, LADA: Latent autoimmune diabetes in adults

### Risk factors for microangiopathy in patients newly diagnosed with EODM

1)The correlations between microangiopathy and clinical characteristics were analyzed. Notably, there were significant differences in the classification of diabetes, age at first treatment, time from onset to first hospitalization, height, UA, UACR, marriage status, macrovascular diseases, peripheral neuropathy and ketosis at onset between the microangiopathy group and the nonmicroangiopathy group (p < 0.05). No difference was found in other aspects (p > 0.05) (Table 2**)**.

**Table 2:** Comparison of microangiopathy in patients with early-onset diabetes

Variables	Microangiopathy group	Nonmicrovascular group	|t/X^2^|	P
**Sex (%)**			0.960	0.378
Male female	44 (6.7) 21 (3.2)	433 (66.1) 157 (24.0)		
**onset age (year)**	31.02 ± 0.76	29.59 ± 0.25	1.808	0.77
**Types of diabetes (%)**			4.217	0.031
T1DM T2DM LADA Special types	5 (0.8) 57 (8.7) 2 (0.3) 1 (0.2)	127 (19.4) 434 (66.3) 22 (3.4) 7 (1.1)		
**Age of first treatment (year)**	31.45 ± 0.77	29.85 ± 0.25	1.993	0.047
**Time from onset to first hospitalization (month)**	7.07 ± 0.95	6.59 ± 0.27	2.153	0.032
**BMI (kg/m** ^**2**^ **)**	26.92 ± 0.82	26.31 ± 0.24	0.791	0.429
**Weight (kg)**	77.01 ± 2.80	77.33 ± 0.79	0.127	0.899
**Height (cm)**	168.48 ± 1.20	170.84 ± 0.33	2.210	0.027
**Initial ketosis (%)**			6.246	0.013
yes no	22 (3.4) 43 (16.9)	296 (45.2) 294 (44.9)		
**Weight loss (%)**			0.355	0.585
yes no	40 (6.1) 25 (3.8)	385 (58.8) 205 (31.3)		
**Smoking status (%)**			0.001	1.000
yes no	27 (4.1) 38 (5.8)	245 (37.4) 345 (52.7)		
**Alcohol (%)**			0.148	0.776
yes no	21 (3.2) 44 (6.7)	177 (27.0) 413 (63.1)		
**DM family history (%)**			1.178	0.298
yes no	37 (5.6) 28 (4.3)	294 (44.9) 296 (45.2)		
**FBG (mmol/L)**	8.39 (6.84,13.45)	9.15 (6.5,13.79)	0.069	0.945
**HbA1c (%)**	9.90 ± 0.36	10.28 ± 0.12	0.983	0.326
**Fatty liver (%)**			1.218	0.638
yes no	39 (6.0) 26 (4.0)	328 (50.1) 261 (39.8)		
**Hypertension (%)**			1.605	0.265
yes no	18 (2.7) 47 (7.2)	123 (18.8) 466 (71.2)		
**Marriage (%)**			4.182	0.042
yes no	49 (7.5) 16 (2.4)	369 (56.3) 221 (33.7)		
**Macrovascular diseases (%)**			10.847	0.002
yes no	21 (3.2) 44 (6.7)	94 (14.4) 496 (75.7)		
**DPN (%)** yes no	11 (1.7) 54 (8.2)	22 (3.4) 568 (86.7)	18.636	0.001
**Initiation of insulin therapy (%)**			0.072	0.889
yes no	43 (16.9) 22 (3.4)	400 (61.1) 190 (29.0)		
**UACR (mg/g)**	58.0(15.5,141.0)	8.00(5.00,13.00)	8.459	0.0001
**SCr (umol/L)**	68.72 ± 3.54	66.59 ± 1.50	0.455	0.649
**TG (mmol/L)**	2.03 (1.20,2.81)	1.57 (1.00,2.63)	1.776	0.076
**TC (mmol/L)**	4.54 (3.78,5.52)	4.41 (3.78,5.23)	0.714	0.475
**HDL-C (mmol/L)**	0.95 (0.79,1.08)	0.94 (0.79,1.15)	0.175	0.861
**LDL-C (mmol/L)**	2.77 (2.38,3.37)	2.80 (2.22,3.33)	0.720	0.472
**ALT (U/L)**	26.10 (17.00,17.90)	26.00 (13.60,44.70)	1.079	0.280
**AST (U/L)**	16.20 (12.60,25.00)	17.90 (13.20,23.80)	1.036	0.300
**GGT (U/L)**	28.20 (16.30,55.20)	25.3 5(14.40,47.20)	1.066	0.286
**UA (umol/L)**	355.5 (302.4,413.4)	324.25 (248.7,410.6)	2.517	0.012

T1DM: Type-1 diabetes mellitus, T2DM: Type-2 diabetes mellitus, LADA: Latent autoimmune diabetes in adults, BMI: body mass index, HbA1c: Glycosylated Hemoglobin, DPN: Diabetic Peripheral Neuropathy, SCr: Serum creatinine, TG: Triglyceride, TC: total cholesterol, HDL-C: high-density lipoprotein cholesterol, LDL-C: low-density Lipoprotein cholesterol, UA: Uric Acid, GGT: gamma-glutamyl transpeptidase, AST: aspartic transaminase, ALT: alanine aminotransferase, FBG: Fasting Blood-glucose, 2hBG: 2 h blood glucose, UACR: Urinary albumin-to-creatinine ratio

2)The results of binary logistic regression analysis demonstrated that high UA, a high UACR and DPN were risk factors for microangiopathy in patients with EODM (Table 3). (A collinearity test was performed on the significant variables, and the results showed tolerance > 0.1 and VIF value < 10 and no collinearity was found between these independent variables.)

**Table 3:** Multivariate analysis of microangiopathy in patients with early-onset diabetes

Variables	r	SE	Wald χ2	P	Exp (B)	(β) 95% CI
**Types of diabetes** ^*^	0.690	0.563	1.501	0.220	1.994	(0.661 ~ 6.013)
**Age of first treatment**	-0.017	0.034	0.242	0.623	0.983	(0.920 ~ 1.051)
**Time from onset to first hospitalization**	-0.001	0.022	0.001	0.980	0.999	(0.957 ~ 1.043)
**UA**	-0.040	0.020	4.102	0.043	0.961	(0.924 ~ 0.999)
**Initial ketosis** ^**#**^	-0.148	0.343	0.186	0.666	0.863	(0.441 ~ 1.689)
**Marriage** ^**#**^	0.549	0.420	1.708	0.191	1.566731	(0.760 ~ 3.940)
**Macrovascular diseases** ^**#**^	0.623	0.366	2.888	0.089	1.864	(0.909 ~ 3.824)
**DPN** ^**#**^	1.424	0.477	8.890	0.003	4.152	(1.629 ~ 10.586)
**Height**	-0.0001	0.001	0.477	0.490	1.000	(0.999 ~ 1.002)
**UACR**	0.015	0.002	38.559	0.0001	1.015	(1.010 ~ 1.020)

*: vs. Type-1 diabetes. #: vs. yes

UACR: Urinary albumin-to-creatinine ratio, UA: Uric Acid, DPN: Diabetic Peripheral Neuropathy

**Fig. 4:**
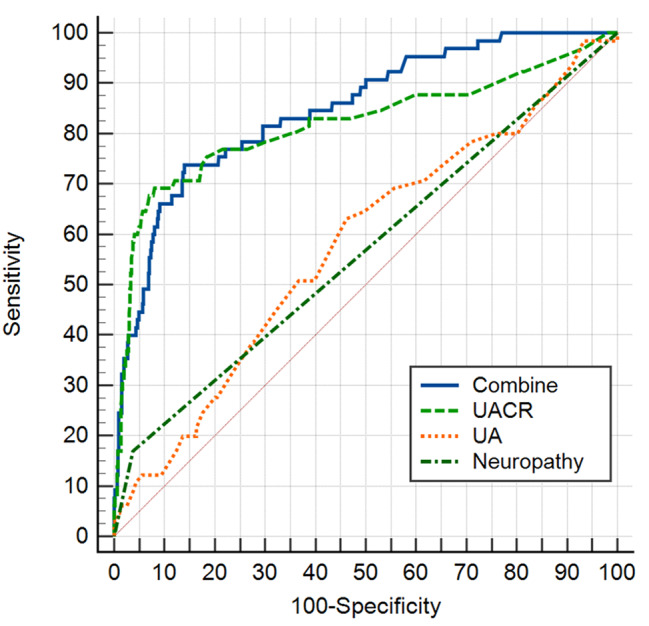
ROC curves for the predictive value of risk factors for microangiopathy in patients with EODM. (The AUC (0.680 (95% CI: 0.643–0.716, sensitivity 49.2%, specificity 78.1%, p < 0.001)) of combined factors had the highest predictive value)

3) To further explore the predictive value of the risk factors (UA, UACR and DPN) of microangiopathy in patients newly diagnosed with EODM, ROC curves were drawn and demonstrated an AUC of 0.848 (95% CI: 0.818–0.875, sensitivity 49.2%, specificity 78.1%, p < 0.001) for combined factors (UA + UACR + DPN) (Fig. 4**)**. It is important to note that the AUC of combined factors had higher predictive value than any single factor.

4) No significant difference was found in HOMA2-%β, HOMA2-IR, HOMA2-%S and C-peptide levels between the microangiopathy group and the nonmicroangiopathy group (p > 0.05) (Table 4).

**Table 4:** Comparison of insulin resistance and insulin secretion function in the two groups of patients

Variables	Microangiopathy group	Nonmicrovascular group	|t/F|	p
LnHOMA2-%β*	3.87 ± 0.12	3.80 ± 0.04	0.176	0.861
LnHOMA2-%S*	3.95 ± 0.10	4.17 ± 0.03	2.437	0.015
LnHOMA2-IR*	0.66 ± 0.10	0.43 ± 0.03	2.449	0.015
** C-peptide%** ^**#**^	29.70 ± 28.70	28.50 ± 1.72	0.13	0.897

#3 patients in the microangiopathy group, 111 patients in the nonmicrovascular lesion group

*62 patients in the microangiopathy group, 479 patients in the nonmicrovascular lesion group

### Risk factors for ketosis in patients newly diagnosed with type 2 EODM

1)The correlations between ketosis and clinical characteristics were analyzed. Notably, there were statistically significant differences in sex, age of onset, DM family history, weight loss at an early stage, HbA1c, HDL-C, GGT, hypertension history and macrovascular diseases between the ketosis group and the nonketosis group in patients with early-onset T2DM (p < 0.05). There were no significant differences in other aspects (p > 0.05) (Table 5**)**.

**Table 5:** Comparison of ketosis in patients with type-2 early-onset diabetes

Variables	Ketosis group	Nonketosis group	|t/X^2^|	P
**Sex (%)**			5.510	0.019
male female	164 (33.4) 37 (7.5)	210 (42.8) 80 (16.3)		
**onset age (year)**	29.59 ± 0.40	31.31 ± 0.34	3.593	0.0001
**BMI (kg/m** ^**2**^ **)**	27.59 ± 0.39	28.55 ± 0.31	1.960	0.053
**Weight (kg)**	82.32 ± 1.26	83.62 ± 1.04	0.799	0.425
**Height (cm)**	171.91 ± 0.56	170.85 ± 0.46	1.466	0.143
**Weight loss (%)**			37.707	0.0001
yes no	152 (31.0) 49 (10.0)	139 (28.3) 151 (30.8)		
**Smoking status (%)**			0.754	0.385
yes no	96 (19.6) 105 (21.4)	127 (25.9) 163 (33.2)		
**DM family history (%)**			5.056	0.025
yes no	100 (20.4) 101 (20.6)	174 (35.4) 116 (23.6)		
**FBG (mmol/L)**	9.38(6.60,13.45)	8.73(6.4,13.38)	1.400	0.161
**HbA1c (%)**	10.88 ± 0.20	9.41 ± 0.14	6.160	0.0001
**Fatty liver (%)**			2.213	0.331
yes no	137 (27.9) 64 (13.0)	210 (42.8) 79 (16.1)		
**Hypertension (%)**			4.218	0.040
yes no	45 (6.5) 156 (31.8)	89 (18.1) 200 (40.7)		
**Macrovascular diseases (%)**			6.212	0.013
yes no	30 (6.1) 171 (34.8)	70 (14.3) 220 (44.8)		
**DPN (%)**			2.230	0.135
yes no	7 (1.4) 194 (39.5)	19 (3.9) 271 (55.2)		
**Microangiopathy (%)**	17 (3.5)	40 (8.1)	3.293	0.070
yes no	184 (37.5)	250 (50.9)		
**SCr (umol/L)**	65.80 (56.45,74.15)	66.60 (58.35,76.45)	1.554	0.120
**UACR (mg/g)**	9.00 (6.00,15.00)	9.00 (5.00,22.00)	0.331	0.740
**TG mmol/L)**	1.86 (1.18,3.01)	2.01 (1.25,3.12)	0.784	0.433
**TC (mmol/L)**	4.54 (3.78,5.52)	4.53 (3.89,5.37)	0.475	0.634
**HDL-C (mmol/L)**	0.86 (0.70,1.01)	0.90 (0.78,1.07)	2.669	0.008
**LDL-C (mmol/L)**	2.89 (2.34,3.42)	2.82 (2.34,3.38)	1.020	0.308
**ALT (U/L)**	26.70 (13.85,55.95)	30.40 (19.68,52.33)	1.458	0.145
**AST (U/L)**	17.70 (12.65,33.10)	19.35 (14.65,28.65)	1.324	0.186
**GGT (U/L)**	27.10 (16.05,56.85)	35.40 (21.38,55.88)	2.223	0.026
**UA (umol/L)**	353.10 (271.50,444.55)	347.95 (279.40,423.35)	0.321	0.748

BMI: body mass index, HbA1c: Glycosylated Hemoglobin, DPN: Diabetic Peripheral Neuropathy, SCr: Serum creatinine, TG: Triglyceride, TC: total cholesterol, HDL-C: high-density lipoprotein cholesterol, LDL-C: low-density lipoprotein cholesterol, UA: Uric Acid, GGT: gamma-glutamyl transpeptidase, AST: aspartic transaminase, ALT: alanine aminotransferase, FBG: Fasting Blood-glucose, 2hBG: 2 h blood glucose, UACR: urinary albumin-to-creatinine ratio

2) The results of binary logistic regression analysis demonstrated that high HbA1c, young age of onset and weight loss at an early stage were risk factors for ketosis in patients with early-onset T2DM (Table 6). (A collinearity test was performed on the significant variables, and the results showed tolerance > 0.1 and VIF value < 10 and no collinearity was found between these independent variables.)

**Table 6:** Multivariate analysis of ketosis in patients with type-2 early-onset diabetes

Variables	r	SE	Wald χ2	P	Exp (B)	(β) 95% CI
**Weight loss** ^**#**^	1.090	0.221	24.226	0.0001	2.974	(1.927 ~ 4.591)
**HbA1c**	0.163	0.040	16.224	0.0001	1.177	(1.087 ~ 1.274)
**DM family history** ^**#**^	-0.435	0.203	4.598	0.32	0.647	(0.435 ~ 0.963)
**Onset age**	-0.045	0.019	5.649	0.017	0.956	(0.921 ~ 0.992)
**Hypertension** ^**#**^	-0.008	0.243	0.001	0.972	1.008	(0.626 ~ 1.625)
**HDL-C**	0.014	0.090	0.024	0.876	1.014	(0.850 ~ 1.211)
**GGT**	0.001	0.002	0.261	0.609	1.001	(0.997 ~ 1.006)
**Sex** ^*^	-0.325	0.252	1.658	0.198	0.723	(0.441 ~ 1.185)
**Macrovascular diseases** ^**#**^	-0.431	0.269	2.563	0.109	0.650	(0.384 ~ 1.101)

#: vs. yes. *: vs. male

GGT: gamma-glutamyl transpeptidase, HbA1c: Glycosylated Hemoglobin, HDL-C: high-density lipoprotein cholesterol

3) There were statistically significant differences in HOMA2-%β, HOMA2-IR and HOMA2-%S between the ketosis group and the nonketosis group (P < 0.05) (Table 7).

**Table 7:** Comparison of insulin resistance and insulin secretion function in the two groups of patients

Variables	Ketosis group	Nonketosis group	|t/F|	p
LnHOMA2-%β*	3.74 ± 0.06	3.97 ± 0.04	2.036	0.001
LnHOMA2-%S*	4.17 ± 0.04	4.03 ± 0.03	0.001	0.015
LnHOMA2-IR*	0.44 ± 0.04	0.57 ± 0.03	0.001	0.016
** C-peptide%** ^**#**^	45.00 (38.60,55.00)	53.10 (26.75,58.50)	1.305	0.812

#6 patients in the ketosis group, 11patients in the nonketosis group

*190 patients in the ketosis group, 284 patients in the nonketosis group

## Discussion

EODM is defined as young-onset diabetes. Despite ongoing debates regarding the diagnostic age of EODM at home or abroad to date, an age of 18 years is widely accepted as the lower boundary with no unified standard for the age ceiling. Using an age of 40 years as the cutoff, population characteristics would be more obvious and clinically significant [17–19]. On average, patients with EODM have prolonged durations of diabetes and suboptimal glycemic control for a longer time than late-onset diabetes, resulting in a higher risk of serious metabolic disorders. Thus, they are more inclined to experience aggressive progression of acute and chronic complications. It was reported [20] that diabetic ketoacidosis (DKA) was a leading cause of death in children and young adults with T1DM, accounting for 50% of all deaths in this population. An epidemiological survey [21] based on 31 provinces in China from 2015 to 2017 showed that the rates of awareness, treatment and control of diabetes were 43.3%, 49.0% and 49.4%, respectively, which were relatively low, indicating that the prevention and management efforts are still needed. Therefore, fully understanding of the clinical characteristics of an onset age between 18 and 40 years patients with different types of newly diagnosed diabetes mellitus is crucial for early and accurate diagnosis and treatment of diabetes, preventing acute and chronic complications and providing scientific management for patients.

From 2012 to 2019, the number of patients newly diagnosed with EODM each year increased and decreased dramatically in 2020 and 2021 in our study, which was mainly attributed to the inconvenience of routine hospital visits caused by the COVID-19 world pandemic. Our study indicated that males were more likely to develop EODM than females. According to a large-scale national study in China [22], the prevalence of diabetes was higher in men than in women (10.6% vs. 8.8%), coinciding with our study. Previous research also [23]found that the incidence, mortality and related complications in male patients for diabetes mellitus were higher than those in females, which was believed to be related to many factors such as environment, socioeconomic status, genetic susceptibility, life pressure and education level. Additionally, it was previously reported [24] that estrogen had a protective effect against EODM in women. Therefore, more attention and resources should be focused on males.

The 2022 ADA guidelines recommend that the age of screening for prediabetes and diabetes be lowered from 45 to 35 years, which highlights the severe situation of the progression of diabetes in young adults around the world [14]. This study demonstrated that T1DM occurred mainly in the 18-24-years-group, whereas T2DM and latent autoimmune diabetes in adults (LADA) had a later onset age, mainly between 25 and 40 years of age. The results were similar to those of Kim et al [25].

T1DM is a disease characterized by chronic autoimmune destruction of pancreatic β-cells. Occuring at any age, it predominantly affects children and adolescents [26]. Nevertheless, as a metabolic disease, T2DM usually develops at ages older than 50 [27]. Accumulating evidence [7] has also revealed that T2DM gradually tends to occur at younger ages and a high incidence rate was in people aged roughly 30–45 years old. LADA is a subtype of T1DM with the same autoimmune pathogenesis. However, early clinical manifestations of LADA are more similar to those of type-2 diabetes [28]. In terms of onset symptoms, our study found that the classic diabetes symptoms (polydipsia, polyuria, hyperphagia, and weight loss) were the most common clinical onset symptoms, followed by elevated blood glucose. Therefore, it is recommended that high-risk populations undergo regular medical examinations to receive timely medical treatment to control blood glucose levels, especially when typical symptoms and atypical symptoms (blurred vision and acute hypoglycemia) are emerging.

Chronic complications, especially vascular lesions, are the most harmful complications, affecting the prognosis and quality of life of patients. With long-term exposure to hyperglycemia, the incidence of microangiopathy in EODM patients has been increasing. A Japanese study [29] on chronic complications in patients with early-onset T2DM found that among 135 patients aged < 35 years who had diabetic retinopathy, 99 developed proliferative retinopathy before the first visit, 81 had diabetic nephropathy at an average age of 31 years, and 32 became blind at an average age of 32 years. In our study, 65 (9.9%) of 655 newly diagnosed EODM patients developed microangiopathy and high UA, high UACR and DPN were found to be the risk factors for microangiopathy in EODM patients. UA promotes oxidation in the human body and is also an inflammatory promoter. High levels of UA could stimulate the renin-angiotensin system, damage vascular endothelial cells and have a proliferative effect on vascular smooth muscle [30]. A growing body of evidence [31–33] has shown that hyperuricemia was a key risk factor for microvascular diseases and diabetes complications, and urate-lowering treatment could ameliorate diabetic microangiopathy. The UACR is one of the important diagnostic criteria for diabetic nephropathy [11, 12], and many studies have found that a high UA (even within the normal range) significantly increased the risk of a high urinary protein excretion rate. Moreover, it has been reported that a high UACR was a marker of endothelial dysfunction and chronic renal disease (CKD), which is closely related to the progression of diabetic retinopathy (DR) and affects the microvascular system of the kidney and retina [34, 35]. Meanwhile, a study by Antonio et al. found that a high UACR significantly increased the prevalence of DR in patients with type-2 diabetes [35]. On the basis of findings from previous studies [36–38], diabetic microangiopathy was related to blood glucose fluctuations, and hypoglycemia could also promote the occurrence of microangiopathy through oxidative stress, inflammation and endothelial dysfunction, as with hyperglycemia. Due to the limitations of retrospective studies, our study failed to collect relevant parameters to assess glucose variability, such as time in range (TIR) and daily mean amplitude of glycemia excursions (MAGE). In addition, our ROC analysis demonstrated that a combination of UA, UACR and DPN could better predict the risk of diabetic microangiopathy in patients newly diagnosed with EODM and efficiently screen potential high-risk patients in clinical practice.

In recent years, a type of T2DM with spontaneous ketosis-prone characteristics, not requiring insulin therapy once the acute phase is over, has attracted attention [39]. The onset symptoms of these particular patients are similar to the severe hyperglycemia and ketoacidosis seen in patients with T1DM. In our study, early-onset T2DM patients with unintentional weight loss, high HbA1c and young onset age were more likely to develop ketosis at the early stage of the diabetes, and these patients typically suffered worse islet function impairment. A study conducted by Zhang [40] on patients with ketosis-prone early-onset T2DM showed that the mean onset age of patients was 28, which was consistent with our study. Although β-cell dysfunction in ketosis-prone T2DM patients is reversible, they more often had higher HbA1c and worse islet function reserve than typical T2DM patients, which are important factors for their susceptibility to ketosis [40–42]. Patients with ketosis-prone early-onset T2DM are more likely to suffer emaciation and weight loss due to poor islet function and high blood glucose, dehydration caused by osmotic diuresis and the inability of peripheral tissues to utilize glucose, causing the human body to dissolve fat and protein. Based on islet autoantibodies and pancreatic function levels, ketosis-prone T2DM can be divided into four subtypes. Unfortunately, we failed to collect all patient’s islet autoantibody status and clinical characteristics of each subtype. In general, with regard to early-onset T2DM, we should pay more attention to the metabolic status of patients with weight loss, high HbA1c and young age of onset to actively control blood glucose and reduce acute complications.

However, our study has several limitations. First, more detailed and comprehensive data associated with EODM patients need to be collected. Second, further randomized trials with a larger sample size should be conducted to confirm our results. Finally, the molecular mechanisms underlying these relationships should be further explored.

To the best of our knowledge, the present study is the first to investigate the clinical characteristics of different types of newly diagnosed diabetes mellitus patients with an onset age between 18 and 40 years. Through a retrospective analysis of 655 patients with EODM, we found that the prevalence of early-onset diabetes has been increasing. Patients with high UA, high UACR and DPN had a propensity for developing diabetic microangiopathy. Comprehensive evaluation of these risk factors could have high value in the prediction, diagnosis and treatment of potential diabetic microangiopathy. For early-onset T2DM, we should pay more attention to the metabolic status of patients with weight loss, high HbA1c and young onset age. More efforts are needed to strengthen screening and management so as to better diagnose and manage EODM, reduce complications and improve quality of life.

## Data Availability

The raw data supporting the conclusions of this article will be made available from the corresponding author upon request and without undue reservation.
